# Assessing the elastic properties of skeletal muscle and tendon using shearwave ultrasound elastography and MyotonPRO

**DOI:** 10.1038/s41598-018-34719-7

**Published:** 2018-11-20

**Authors:** Y. N. Feng, Y. P. Li, C. L. Liu, Z. J. Zhang

**Affiliations:** 1 Luoyang Orthopedic Hospital of Henan Province, Orthopedic Hospital of Henan Province, Luoyang, China; 2 Clinical College of Acupuncture, Moxibustion and Rehabilitation, Guangzhou University of Chinese Medicine, Guangzhou, China

## Abstract

The purposes of this study were to compare Young’s modulus values determined by shear wave ultrasound elastography (SWUE) with stiffness index obtained using a hand-held MyotonPRO device on the resting stiffness of gastrocnemius muscle belly and Achilles tendon; and to examine the test-retest reliability of those stiffness measurement using hand-held MyotonPRO. Twenty healthy volunteers participated in the study. The measurement values of muscle and tendon was determined in dominant legs. Each marker point was assessed using MyotonPRO and SWUE, respectively. Intra-operator reliability of MyotonPRO was established in 10 of the subjects. The correlation coefficients between the values of muscle and tendon stiffness indices determined by MyotonPRO and SWUE were calculated. Significant correlations were found for muscle and tendon stiffness and Young’s modulus ranged from 0.463 to 0.544 (all *P* < 0.05). The intra-operator reliability ranged from good to excellent (ICC_(3,1)_ = 0.787~0.928). These results suggest that the resting stiffness of gastrocnemius muscle belly and Achilles tendon measured by MyotonPRO is related to the Young’s modulus of those quantified by SWUE. The MyotonPRO shows good intra-operator repeatability. Therefore, the present study shows that MyotonPRO can be used to assess mechanical properties of gastrocnemius muscle belly and Achilles tendon with a resting condition.

## Introduction

The human gastrocnemius muscle belly and Achilles tendon exhibit spring-like characteristics during running and hopping^1,2^. The stiffness of muscle and tendon represents tissue mechanical properties on the longitudinal axis as measured by tissue resistance during passive stretching^3,4^. It is mainly responsible for transmitting and absorbing energy of motion in various sports activities^5^. Moreover, the stiffness of muscle and tendon could indicate tissue condition objectively^6,7^, such as pain^8^, fatigue^9^ and cramps^10,11^. A recent study conducted by Kalkhoven *et al*.^12^ found that higher stiffness of the medial gastrocnemius muscles appears to be beneficial to athletic performance for football players. Thus, it is important to accurately quantify the stiffness of the medial gastrocnemius (MG) and lateral gastrocnemius (LG) muscles as well as the Achilles tendon (AT) for monitoring the changes of the stiffness of muscle and tendon.

Recently, shear wave ultrasound elastography (SWUE), has been used as a non-invasive and objective measure of muscle and tendon stiffness^13–18^. SWUE evaluates the tissue stiffness as Young’s modulus by analyzing propagation velocity of induced shear waves from an entire defined region of interest (ROI) in the targeted muscle and tendon. Our previous studies have demonstrated that SWUE is a valid and reliable tool to quantify the elastic properties of the healthy tendon^16,18,19^, pathological tendon^8^ and muscle^20^. In addition, the SWUE has been validated for estimating the elastic properties of skeletal muscles using materials testing technique^4^. The results from other studies have revealed that the SWUE can quantify the elastic properties of various skeletal muscles^21–23^ and tendons^24,25^. Although SWUE is a valid and reliable tool for quantifying the stiffness of muscle and tendon, it costs and required technical expertise limit wider clinical use.

Access to a SWUE may be limited at large hospital and research institute. Procuring a SWUE is not always feasible in most clinics due to it is expensive and maintenance cost. A portable device equipped with the stiffness of muscle and tendon measurement function has a comparatively lower cost that is applicable for most small-scale clinics and research laboratory. More recently, a new hand-held device, called MyotonPRO, provides a reliable, accurate and sensitive way for the objective and non-invasive digital palpation of superficial skeletal muscles^26,27^. It enables measurement not only of muscles^27^, but also tendons^28^. Thus, MyotonPRO may become a basic evaluation technique in muscle and tendon assessment as a portable and convenient diagnostic and monitoring device in medical practice^29–31^. Thus, it is important to examine the inter-equipment variability between SWUE and hand-held MyotonPRO for accurately quantifying the stiffness of the resting stiffness of gastrocnemius muscle belly and Achilles tendon. No investigation has been conducted to examine the correlation between the Young’s modulus on gastrocnemius muscle belly and Achilles tendon captured from SWUE and the stiffness index obtained from the MyotonPRO device.

The aims of the present study were (1) to assess the correlation of the stiffness index of gastrocnemius muscle belly and Achilles tendon computed from an hand-held device MyotonPRO and Young’s modulus from a SWUE; and (2) to determine test-retest reliability of measuring the stiffness of gastrocnemius muscle belly and Achilles tendon using MyotonPRO and to determine the minimal detectable change (MDC).

## Methods

### Ethics statement

The present study was approved by the Human Subjects Ethics Committee of the Clinical Medical College of Acupuncture, Moxibustion, and Rehabilitation, Guangzhou University of Chinese Medicine. The experimental procedures were conducted in conformity with the Declaration of Helsinki. The procedures of the study were fully explained to the participants and they provided their informed written consent before testing.

### Participants

Twenty healthy volunteers (mean age: 22.8 ± 3.7 years; mean height: 167.9 ± 8.1 cm; mean body mass: 60.0 ± 9.9 kg) were recruited for this study. Exclusion criteria consisted of body mass index (BMI) > 30 kg/m^2 ^^32^ (BMI was defined as the body mass divided by the square of the body height), past or current neuromuscular disease, musculoskeletal injury and current pain in the lower limbs.

### Experimental setup and protocol

Prior to the measurement, subjects performed a self-paced walking warm-up for 5 min. The procedures of testing were the same as those used in the study of Kelly *et al*.^32^. The following measurements were made while subjects lay prone on a bed with knee extension and the ankle in a neutral position using a self-made ankle foot orthosis. The Young’s modulus and stiffness of the gastrocnemius and Achilles tendon in dominant leg was quantified in this study. The dominant leg was determined by asking the subject which leg he or she preferred to use in kicking a ball^33^. All tests were performed in the afternoon in independent quiet spaces with an average indoor temperature of 25 °C.

### Subject’s position

The skin over the gastrocnemius muscle belly and Achilles tendon area was exposed for testing. The location for stiffness measurement was identified and marked with a permanent marker. Stiffness of MG and LG was measured at 30% of the lower leg length from the fibulae capitulum or proximal medial condyle of tibia to Achilles tendon insertion site where almost the maximal cross-sectional area in the lower leg is observed^3,34^. For MG and LG measurement, the ultrasound transducer was transversely positioned on the muscle belly and then move from medial to lateral direction. The highest muscle thickness was observed in the image and then the area was marked on the skin for the MyotonPRO device and SWUE measurement. The AT was tested 5 cm above the tuber calcanei^28^.

#### Hand-held MyotonPRO measurements for muscle and tendon

**Figure 1:**
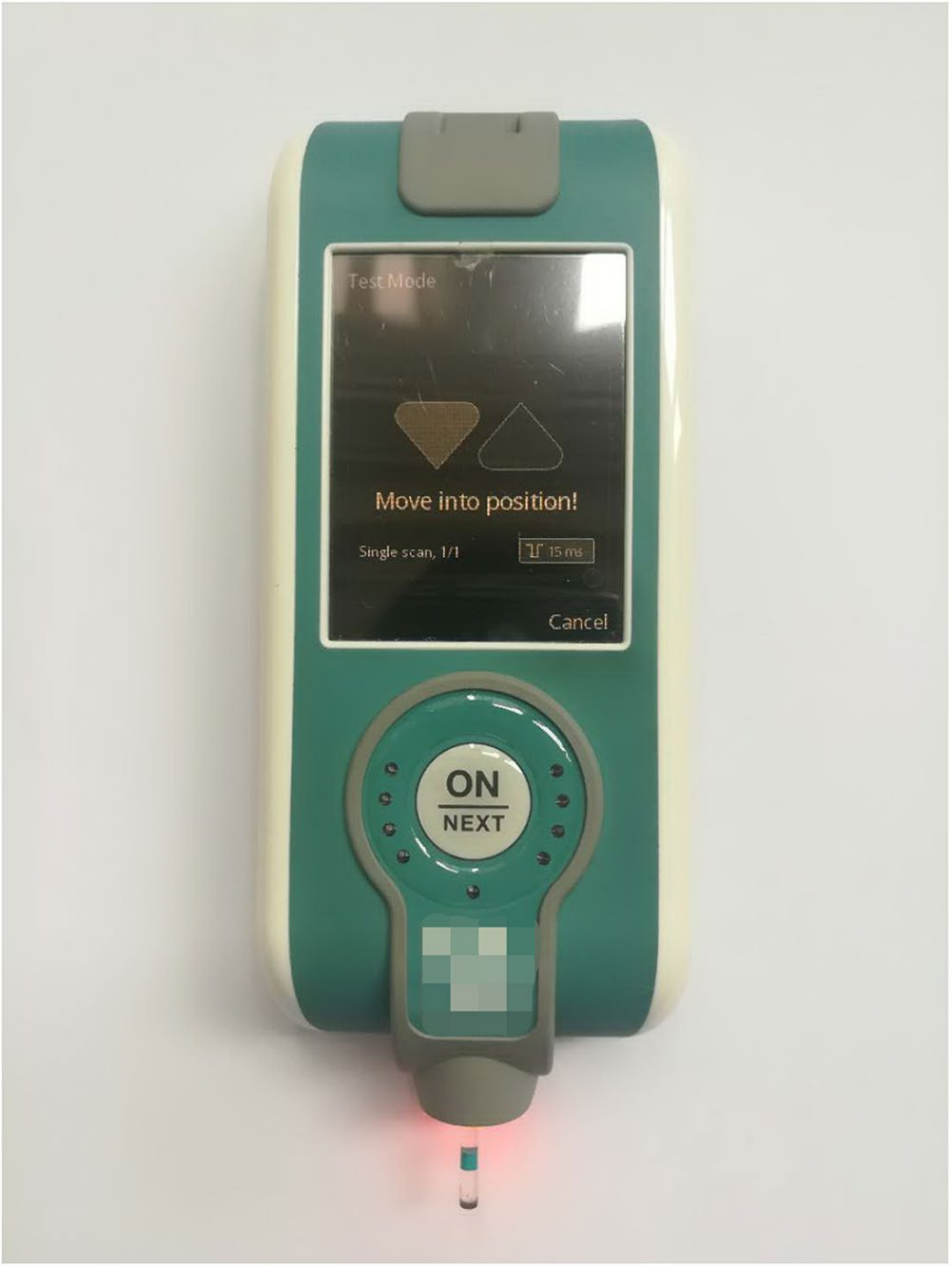
The photograph of a MyotonPRO device.

The MyotonPRO (Myoton AS, Tallinn, Estonia) is a small, handheld and digital palpation device (Fig. 1). The stiffness of gastrocnemius muscle-tendon unit is a biomechanical property that characterizes resistance to a contraction or to an external force that deforms its initial shape. The device provides a controlled preload of 0.18 N for initial compression of the subcutaneous tissue and then release an additional 15-ms impulse of 0.40 N of mechanical force, which induces a damped or decaying natural oscillation of the tissue^35^. The calculation formula was as follows: S = a_max_. m_probe_/Δl; a_max_ - maximum amplitude of the oscillation in the acceleration signal; m_probe_ - mass (preload) of the probe of 0.18 N, Δl - amplitude of the displacement signal. To determine intra-operator reliability between sessions, ten of the subjects (mean age: 22.5 ± 2.9 years; mean height: 168.2 ± 6.4 cm; mean body mass: 58.0 ± 5.4 kg) returned 5 days later and the same testing procedure was repeated by the original operator. For this study, three measurements on each tested point on the muscle and tendon was used and the mean was calculated for further analysis.

#### SWUE measurements for muscle and tendon

**Figure 2:**
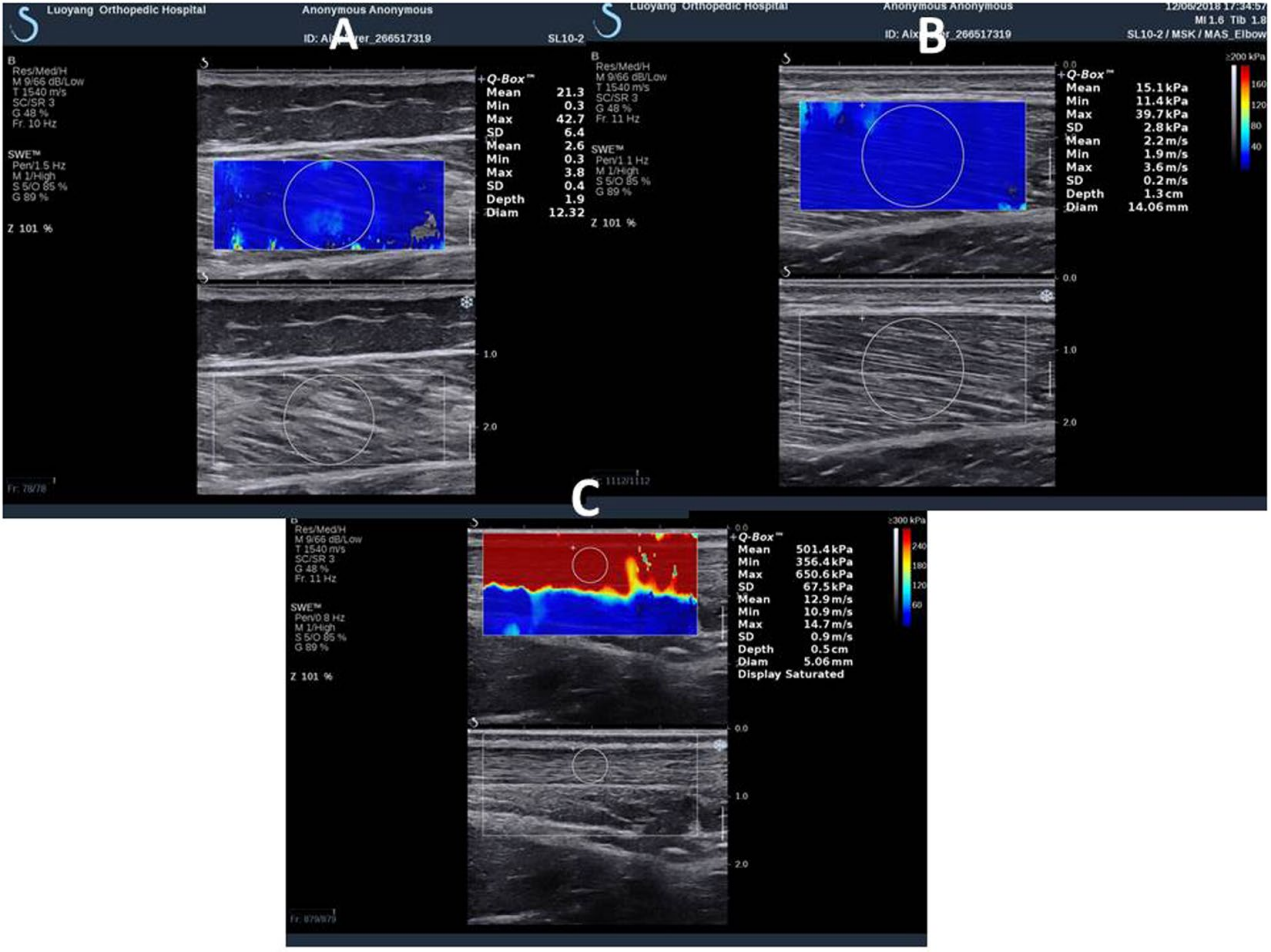
Representative images for shear modulus measurement using shear wave ultrasound elastography: (**A**) lateral gastrocnemius (LG) (**B**) medial gastrocnemius (MG) muscles belly, (**C**) Achilles tendon (AT). The figure includes ultrasound B-mode images of LG (**A**), MG (**B**) and AT (**C**) in the sagittal plane (red color indicates hardness; green color indicates soft). The scale for the color code is provided to the right as estimated shear modulus. The spatial average of shear modulus in a circular area was calculated.

The mechanical properties of the gastrocnemius muscle-tendon unit were measured using SWUE technology with an Aixplorer® ultrasound unit (Supersonic Imaging, Aix-en-Provence, France) equipped with a 4–15 MHz linear-array transducer. The parameters of SWUE were adopted our previous studies^18,20^. The musculoskeletal acquisition mode was used to measure the Young’s modulus of the muscle and tendon with the temporal averaging (persistence) and spatial smoothing set to medium and six, respectively. The ultrasound transducer was positioned at the marked regions, with light pressure on top of a generous amount of coupling gel. The transducer was kept stationary for 8–12 s during the acquisition of the SWE sonogram. The elasticity map was selected about 8–12 s^18,20^. A total of 3 images were captured for muscle and tendon on each marked region for off-line analysis. For measuring the stiffness of the LG and MG, the conventional grey scale of the SWUE was used to identify the muscles (LG and MG). Once the muscle was identified, the transducer was aligned and parallel to muscle fibers and SWE mode was activated to quantify the shear elastic modulus of muscle. A circle delineating the Q-box was centered on the targeted muscle including LG (Fig. 2A) and MG (Fig. 2B). The diameter of Q-box was defined by the thickness of the muscle, which was determined by the distance between the superficial and deep muscle fasciae. For quantifying the Young’s modulus of Achilles tendon, the transducer was located on the 5 cm above the tuber calcanei^28^. B-mode was used to locate and align the Achilles tendon longitudinally with the probe of ultrasound unit. When a clear image of Achilles tendon was captured, the shearwave mode was activated. The region of interest (ROI) was covered on the targeted muscle and tendon. The Q-box was located in the center of ROI. The diameter of Q-box was defined by the thickness of Achilles tendon (Fig. 2C). In our unpublished paper, 20 healthy subjects were recruited in this study. The Young’s modulus of MG, LG and AT was quantified using the SWUE. The Intra-operator reliability of Young’s modulus of the MG, LG and AT was 0.91, 0.95 and 0.93. For the inter-operator reliability of the MG, LG and AT was 0.89, 0.91 and 0.89.

#### Statistical analyses

SPSS (version 16.0, SPSS Inc., Chicago, IL) was used for statistical analyses. Descriptive data are presented for all demographic data. To investigate the relationship between values of stiffness determined with MyotonPRO and Young’s modulus obtained by SWUE, Pearson’s product-moment correlation coefficients were calculated at each measurement site. Linear regression analysis was performed with SWUE_measurement as a dependent variable and MyotonPRO_measurement as independent variables. Intraclass correlation coefficient (ICC_3,1_) model 3 were used to evaluate the intra-operator reliability^36^. All reliability coefficients were interpreted as follows: below 0.499 as poor, 0.500 to 0.699 as moderate, 0.700 to 0.899 as good, and 0.900 to 1.000 as excellent^37^. The standard error measurement (SEM) was computed (using the formula SEM = standard deviation ×√1-ICC). Afterward, a Bland-Altman plot was constructed to further evaluate the reliability of MyotonPRO technique, which measure bias between measurement methods as well as variability of scatter^38,39^. Bland and Altman analysis was used to identify the systematic error^40^. An alpha of 0.05 was used to set the level of statistical significance. The data are presented as mean ± standard deviation in the text.

## Results

### Relationship between MyotonPRO and SWUE for measurement of muscle and tendon stiffness

**Figure 3:**
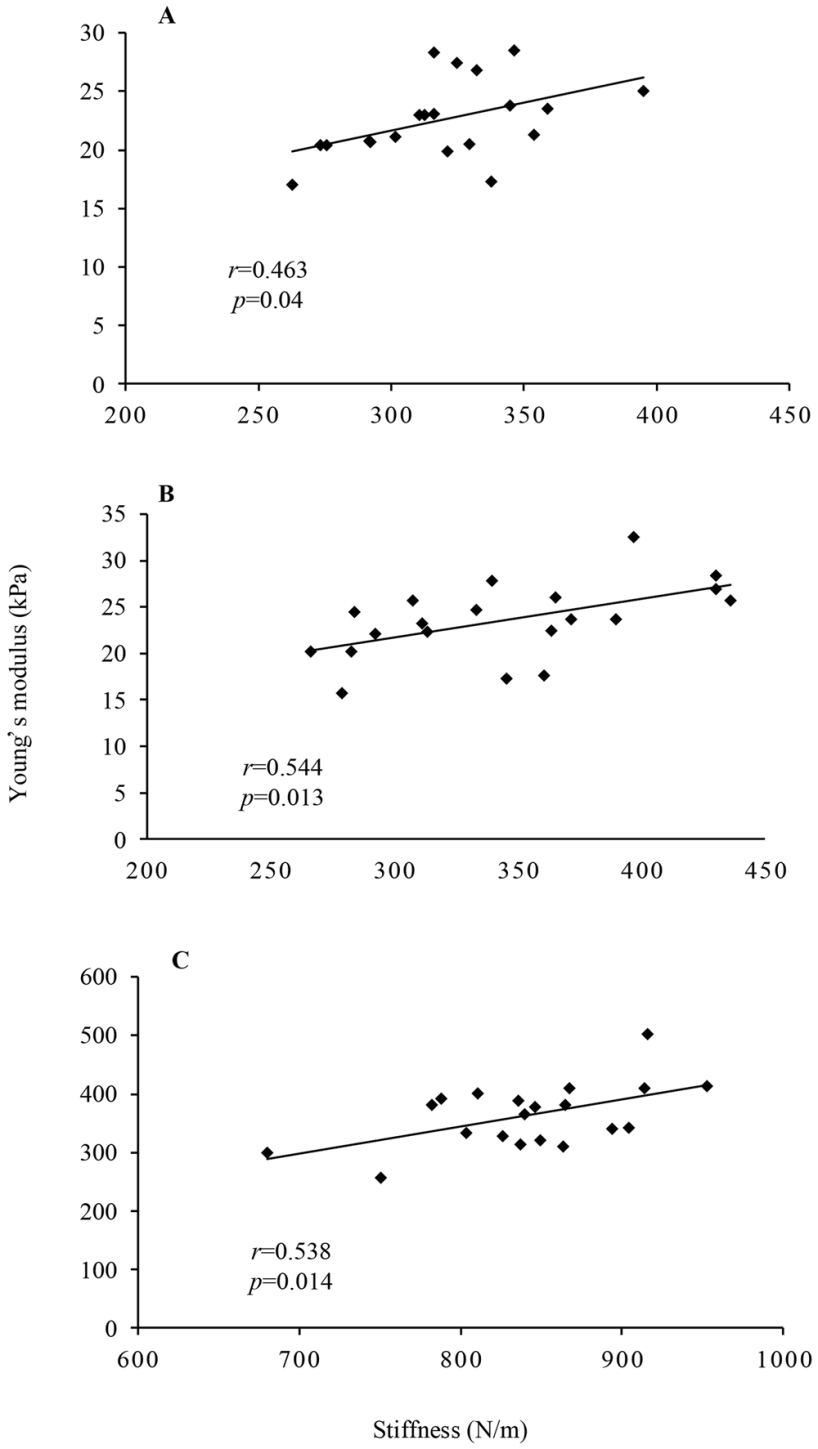
Correlations between stiffness and the Young’s modulus of muscle and tendon obtained from MyotonPRO and SWUE, respectively. (**A**) The medial gastrocnemius muscle; (**B**) the lateral gastrocnemius muscle; (**C**) the Achilles tendon.

**Figure 4:**
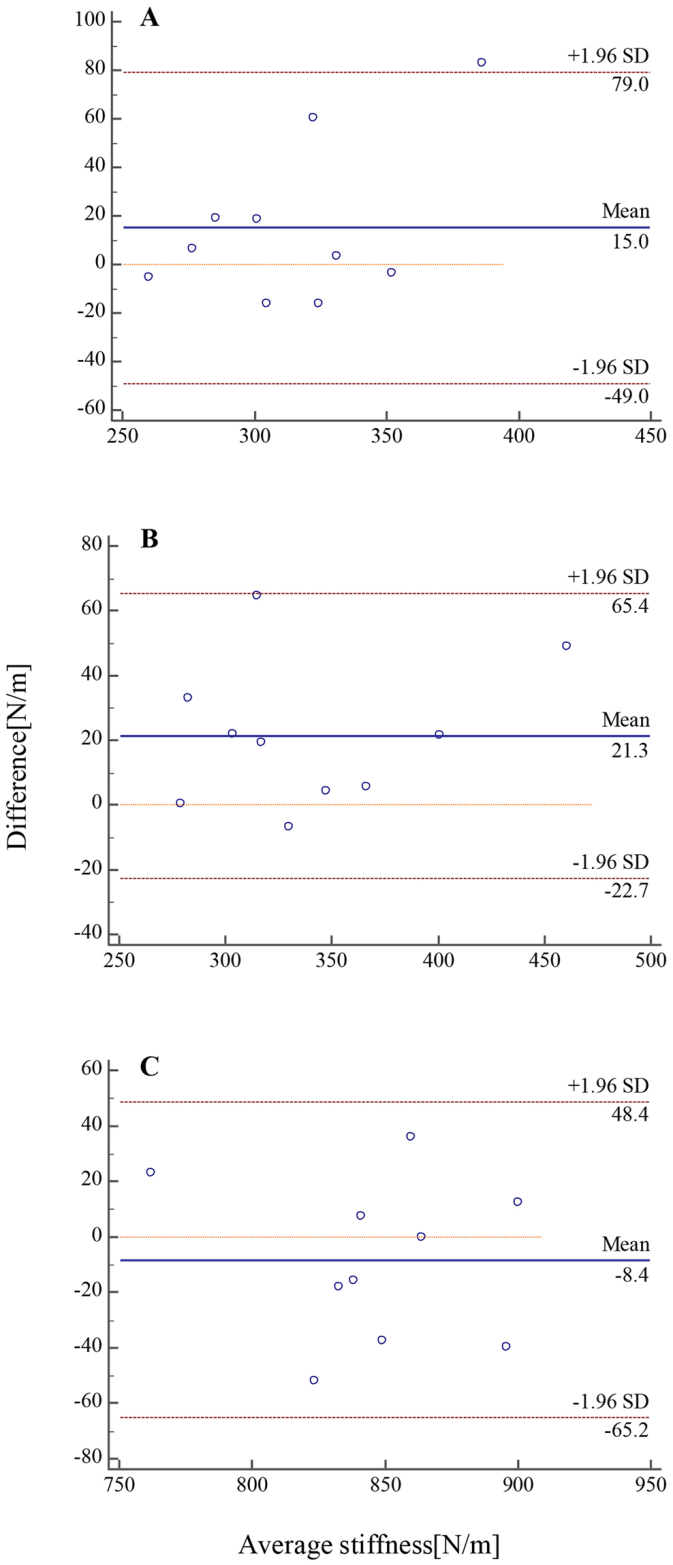
Bland-Altman plot showing mean differences in measurement of gastrocnemius muscle belly and Achilles tendon stiffness in dominant legs between 5 days by the same operator. (**A**) Stiffness of the medial gastrocnemius muscle, (**B**) stiffness of the lateral gastrocnemius muscle, (**C**) stiffness of the Achilles tendon. Broken lines indicate the 95% limits of agreement.

A significant correlation was found between the values of the gastrocnemius muscle belly and Achilles tendon stiffness indices determined with MyotonPRO and Young’s modulus determined with SWUE (MG: *r* = 0.463, *p* = 0.040; LG: *r* = 0.544, *p* = 0.013; AT: *r* = 0.538, *p* = 0.014) (Fig. 3B) (Table 1). From linear regression model, the equation: SWUE_measurement = −26.4 + 0.46 × MyotonPRO_measurement (*R*^2^ = 0.289; *P* = 0.014) for Achilles tendon; for MG: SWUE_measurement = 7.37 + 0.48 × MyotonPRO_measurement (*R*^2^ = 0.214; *P* = 0.04); for LG: SWUE_measurement = 9.19 + 0.42 × MyotonPRO_measurement (*R*^2^ = 0.295; *P* = 0.013). This result validated the hypothesis that Young’s modulus of the gastrocnemius muscle belly and Achilles tendon evaluated by SWUE corresponds to those stiffness evaluated with MyotonPRO to some extent (Fig. 4).

**Table 1:** Correlation coefficient between stiffness and the Young’s modulus of muscle and tendon obtained from MyotonPRO and SWUE, respectively.

	Mean ± standard deviation		*r*	*P*
	MyotonPRO(N/m)	SWUE(kPa)		
MG	319.84 ± 32.18	22.59 ± 3.31	0.463	0.040
LG	344.82 ± 53.26	23.56 ± 4.08	0.544	0.013
AT	841.23 ± 62.83	363.38 ± 54.11	0.538	0.014

MG, the gastrocnemius medialis; LG, the gastrocnemius lateralis; AT, Achilles Tendon; SWUE, shear wave ultrasound elastography.

### Intra-operator reliability of MyotonPRO in measuring muscle and tendon stiffness

The muscle and tendon stiffness index in dominant legs is presented in Table 2. The intra-operator reliabilities for the measurements of LG were excellent. The intra-operator reliability value for ICC was 0.928 and for MDC was 48.09 N/m as well as 95% CI was 0.729~0.982, corresponding to an SEM of 17.35 N/m. With regard to the intra-operator reliability of MG and AT, the ICC value was good (ICC_MG_ = 0.787; ICC_AT_ = 0.864) and the MDC value was 28.19 and 34.95 N/m as well as 95% CI was 0.195~0.946 and 0.488~0.966, corresponding to an SEM of 10.17 and 12.61 N/m, respectively.

**Table 2:** The intra-operator reliability of MyotonPRO in measurement of muscle and tendon stiffness.

	Mean ± standard deviation(N/m)		SEM	ICC	95%CI	MDC
	Operator1	Operator2				
**Dominant legs**						
MG	306.80 ± 32.16	321.83 ± 47.74	10.17	0.787	0.195–0.946	28.19
LG	329.50 ± 54.85	350.83 ± 59.97	17.35	0.928	0.729–0.982	48.09
AT	850.79 ± 43.36	842.40 ± 39.88	12.61	0.864	0.488–0.966	34.95

MG, the gastrocnemius medialis; LG, the gastrocnemius lateralis; AT, Achilles Tendon; SEM, standard error mean; ICC, intraclass correlation coefficient; MDC, minimum detectable change; 95%CI, 95% confidence interval.

The Bland-Altman plot for muscle and tendon stiffness shows the bias line, or the mean difference in MG stiffness between 5 days was 15.0 N/m and the 95% limit of agreement was −49.0 to 79.0 N/m. The Bland-Altman plot shows no systematic bias, as the data points distribute equally below and above the mean. The first measure seems to overestimate at stiffness index, with an outliner at the first highest mean stiffness index (Fig. 4A). The bias line, or mean difference in the LG stiffness between 5 days was 21.3 N/m and the 95% limit of agreement was −22.7 to 65.4 N/m. The Bland-Altman plot shows no systematic bias, as the data points distribute equally below and above the mean. (Fig. 4B). The bias line, or mean difference in AT stiffness between 5 days was −8.4 N/m and the 95% limit of agreement was −65.2 to 48.4 N/m. The Bland-Altman plot for Achilles tendon shows no systematic bias, as the data points distribute equally below and above the mean (Fig. 4C).

## Discussion

The present study was designed to compare values of a hand-held tool of measuring the gastrocnemius muscle belly and Achilles tendon stiffness using MyotonPRO with Young’s modulus values determined by SWUE and to verify the correspondence between techniques as well as to evaluate intra-operator reliability of MyotonPRO in measuring those stiffness. Our results indicate a high level of agreement in the gastrocnemius muscle belly and Achilles tendon stiffness measured using MyotonPRO compared with measurements from SWUE. The results also demonstrate good to excellent intra-operator reliability (ICC = 0.737~0.928) between sessions using this technique of MyotonPRO.

### Correlation between MyotonPRO and SWUE for measurement of muscle and tendon stiffness

There is a need to develop easily accessible and cost-effective clinical tools to assess the health of tissue mechanical properties. Thus, we undertook a study to better understand how well digital palpation device (MyotonPRO) measurements associated with SWUE measurements. Our findings were that both the gastrocnemius muscle belly and Achilles tendon stiffness indices determined with MyotonPRO were strong associated with Young’s modulus determined with SWUE. In our previous study, Zhang *et al*.^16^ demonstrate that the shear elastic modulus on the patellar tendon captured from a SWUE is related to the tangent traction modulus quantified by a material testing system. Meanwhile, the SWUE presents good intra- and inter-operator repeatability. Furthermore, the SWUE has also been validated for assessing the stiffness of skeletal muscle. A significant correlation was found between Young’s modulus from the SWUE and measurements from the materials testing system^4^. A recent study published by Kelly *et al*.^32^ compare measurement methods of tissue stiffness using SWE and MyotonPRO in the infraspinatus, erector spinae, and gastrocnemius muscles. The results demonstrate that correlation of the two methods in the three muscle regions were significant (*r* = 0.23–0.71, *p* < 0.05). Yanagisawa *et al*.^7^ compared the findings of ultrasound real-time tissue elastography with those of a tissue hardness meter for semi-quantitative assessment of the hardness of exercised muscles. The results revealed ultrasound real-time tissue elastography was similar to the muscle hardness meter. Ariji *et al*.^41^ investigated the relationship between the masseter muscle elasticity index ratio obtained by sonographic elastography and the hardness measured by a hardness meter in healthy volunteers. The results showed the masseter muscle elasticity index ratio was significantly correlated with the masseter muscle hardness. Moreover, Akagi *et al*.^42^ determined neck and shoulder stiffness values using SWUE and a muscle hardness meter. There were not significant between the values of muscle hardness indices. However, individuals’ subjective neck and shoulder stiffness did not correspond to their objective symptoms. Therefore, the use of SWUE is essential to more precisely assess neck and shoulder stiffness.

SWUE produces elastography images, based on the combination of a focused ultrasound radiation forces and an ultrafast ultrasound acquisition imaging system is capable of capturing the propagating wave in real time, to estimate material properties of a localized area of soft. In recent years, muscles and tendons elasticity has widely been quantified using SWUE^15,17^. Drakonaki *et al*.^43^ obtained moderate to good intra and inter-operator reliability (ICC = 0.51~0.78) in assessing the stiffness of Achilles tendons. In a study by Brandenburg *et al*.^44^, the reliability of measurements was good to excellent (ICC = 0.67~0.80), which were in line with the previous studies^4^. Taniguchi *et al*.^45^ reported that shear modulus decreased (*p* < 0.01) by 14% immediately after stretching, compared with before stretching across muscle heads. In 20 min after stretching, the decrease in shear modulus returned. In the measurement of supraspinatus muscle, Itoigawa *et al*.^46^ divided the supraspinatus muscle into four anatomical regions: anterior superficial, posterior superficial, anterior deep, and posterior deep. SWUE combined with B-Mode ultrasound imaging was used to explore muscle stiffness and extensibility in each region. The results demonstrated SWUE combined with B-Mode ultrasound imaging could be a feasible method for quantifying the local stiffness of the rotator cuff muscles. Eby *et al*.^47^ found that sex and age were significant parameters for older adults (>60 years) in full extension. Young’s modulus values increased with advancing age. However, shear modulus values for females tended to be higher than those for males.

### Intra-operator reliability of MyotonPRO in measuring muscle and tendon stiffness

It has been reported that the MyotonPRO is a reliable method for evaluating the mechanical properties of muscles and tendons. The findings of the present study are in accordance with the results of the previous studies. For example, our team revealed excellent intra and inter-tester reliability (ICC = 0.97) for measuring upper trapezius stiffness using hand-held MyotonPRO^26^. Aird *et al*.^48^ reported that repeated measurements had very high within-day (ICC > 0.90) and high between-day (ICC > 0.70) reliability for quadriceps in healthy older males. Fröhlich-Zwahlen *et al*.^27^ examined the test-retest reliability of MyotonPRO to quantify the stiffness of the vastus lateralis, rectus femoris, biceps femoris, tibialis anterior and gastrocnemius medialis stiffness among patients with chronic stroke. The ICC values ranged from 0.77 to 0.92. In a study by Van Deun *et al*.^49^, the stiffness of biceps brachii was quantified using the MyotonPRO. The intra- and inter-rater reliability was high to very high in the healthy subpopulations. In individuals with paratonia, the intra- and inter-rater reliability ranged from low to high. The results of the present study were in line with a recent study^50^. We found ICC values ranged from 0.737 to 0.928.

To date, limited studies have been conducted to compare the 95% limits of agreement. The Bland-Altman plot is a visual presentation that allowed identification of systematic error. There was no systematic error in the muscle and tendon measurements, which indicated consistency between the two measurements between-days. Furthermore, a possible reason for the mean value differs from 0 may be attributed to the changes of muscle tone between-days due to physical activity. It is difficult to control physical activity of subject between-days, which may result in the changes of muscle tone measurement. The findings from the present study provide the reference for quantifying variation in the muscle stiffness after intervention.

There are a few studies reporting the reliability of tendon stiffness measured by MyotonPRO. Nevertheless, some researchers have evaluated tendon stiffness using the MyotonPRO. A study measured by Sohirad *et al*.^51^ found that the reliability of stiffness values was excellent both for the patellar tendon (ICC = 0.96) and the Achilles tendon (ICC = 0.96) in healthy adults of normal body mass index. Meanwhile, the results demonstrate that men had stiffer tendons than women (*p* < 0.05). Pożarowszczyk *et al*.^28^ reported that Achilles tendon stiffness for the dominant leg increased significantly from before fights (751.57 ± 123.493 N/m) to immediately after fights (809.43 ± 160.43 N/m). Schneider *et al*.^35^ had monitored the mechanical properties of skeletal muscles and tendons in weightlessness during parabolic flights.

Clinicians identify the stiffness of the gastrocnemius muscle belly and Achilles tendon mainly on the basis of manual palpation. With the development of technology, the devices for quantifying the stiffness of soft tissues were developed. The SWUE has an advantage in that it is simple to use, virtually real time and mobile. However, a light touch on the skin with the ultrasound probe is needed, which easily cause transverse tissue displacement. Manual compression may alter the mechanical properties of the testing tissues. In contrast, MyotonPRO provides a unique, reliable, accurate and sensitive way for the objective and non-invasive digital palpation of superficial skeletal muscles. But MyotonPRO cannot be used for the measurement of thin muscles, deep muscles and muscles covered by subcutaneous fat (>20 mm). Therefore, qualitative assessment method of tissue mechanical properties should be selected based on the target muscle-tendons.

### Limitations

Limitations of this study should be acknowledged. Firstly, the methods of this study did not investigate the inter-operator reliability of MyotonPRO in measuring the gastrocnemius muscle belly and Achilles tendon stiffness. The day-to-day repeatability of both the SWUE has not been conducted in this study. Secondly, the study included solely healthy younger subjects and therefore, our findings could not be generalized to the whole population (such as healthy elderly); as well as other muscles (such as biceps brachii) and tendons (such as patellar tendon). Then, myoelectrical activity was concurrently not recorded with surface electromyography monitoring to ensure that the gastrocnemius muscle belly remained a resting condition. In addition, correlation SWUE and MyotonPRO for measurement of the gastrocnemius muscle belly and Achilles tendon mechanical properties should be analyzed during active passive ROM.

## Conclusion**s**

In summary, the present study demonstrates that the gastrocnemius muscle belly and Achilles tendon stiffness measured by MyotonPRO is related to the Young’s modulus of those muscle and tendon quantified by SWUE. The MyotonPRO shows good intra-operator repeatability. Therefore, the present study shows that MyotonPRO can be used to assess mechanical properties of the gastrocnemius muscle belly and Achilles tendon with a resting condition.

## Data Availability

All data included in this study are available upon request by contact with the corresponding author.
